# Overall explanation of auxin mechanisms that control vascular differentiation in leaves and organ development in flowers

**DOI:** 10.1007/s00425-025-04716-y

**Published:** 2025-05-15

**Authors:** Roni Aloni

**Affiliations:** School of Plant Sciences and Food Security, Tel Aviv University, Tel Aviv, Israel

**Keywords:** Auxin (free, total), Apical dominance, Anther (tapetum), Hydathode, Leaf morphogenesis, Patterned vascular differentiation, Synchronized flower development

## Abstract

**Main conclusion:**

This review on auxin control mechanisms explains the general concept of *apical dominance* in leaves, flowers and roots, where specific cells or organs that produce high-auxin concentrations inhibit other adjacent tissues or organs, resulting in organized developmental patterns, e.g., the downward (basipetal) development of leaves, organized vein patterns in leaves, synchronized flower development, and optimized root architecture.

**Abstract:**

The various control mechanisms and roles of auxin during leaf and flower development were investigated in the pioneering work of Aloni et al. (Planta 216:841–853, 2003; Planta 223:315–328, 2006a), which explained why and how leaves, flowers and their vascular tissues are regulated in organized patterns. The first paper (Aloni et al. 216:841–853, 2003) tested the *leaf venation hypothesis* (Aloni, J Plant Growth Regul 20:22–34, 2001) and the second paper (Aloni et al. Planta 223:315–328, 2006a) uncovered the unsolved mystery of floral organ developmental pattern. In this review, the precedence and unique contribution of these studies in explaining the general auxin mechanisms controlling vascular differentiation in leaves and organ development in flowers are presented in conjunction with later work that detailed specific aspects of these mechanisms.

## Introduction

Vascular tissue patterning needs to be regulated to produce a functioning transport system. In leaves, the molecular and physiologic mechanisms that control and determine venation pattern formation were poorly understood (for discussion and references see Aloni 2001). Therefore, I suggested the *leaf venation hypothesis* (Aloni 2001) proposing the mechanism of *apical dominance* (Thimann and Skoog 1933) for explaining leaf development and vein pattern formation, as a working hypothesis for those interested in studying vascular differentiation in leaves.

*Apical dominance* is the control exerted by the apical bud that prevents the outgrowth of lateral buds (Thimann and Skoog 1933); the apical growing bud regulates shoot growth by producing high-auxin concentration that controls shoot development by inhibiting the growth of the axillary buds. I proposed that this concept of auxin mechanism might be general and could also explain the control mechanism of additional developing plant organs, like differentiating leaves (Aloni 2001).

In flowers, based on double and triple mutants of *Arabidopsis* homeotic genes, the *ABC model of flower development* was proposed (Bowman et al. 1991), explaining the genetic mechanism how the floral organs: sepals, petals, stamens and carpels are induced. However, although the petal and stamen primordia in flowers appear simultaneously (early stage 5 of flower development), the stamens develop first (during stages 6–8), whereas the early induced petal primordia do not grow until stage 9 (Bowman et al. 1989), which is a phenomenon that has wide occurrence among flowering plants (Endress 1994).

To solve this mystery, we have tried to understand the hormonal mechanism that controls this floral organ pattern, by clarifying the possible role of auxin in flower development (Aloni et al. 2006a). The idea to study the possible involvement of auxin in flower development was promoted by the study on the pin-formed mutant pin1-1 that has several structural abnormalities in inflorescence axes, flowers, and leaves, which suggested that the normal level of polar auxin transport activity, regulated by the PIN1 gene, is required in early developmental stages of floral bud formation in *Arabidopsis* (Okada et al. 1991).

## Patterns of auxin production and vascular differentiation in leaves

Our study (Aloni et al. 2003) aims to elucidate, with various molecular tools, where free auxin (the bioactive hormone, Indole-3-acetic acid, IAA) is produced in dicotyledonous leaves. The objective was to clarify how fundamental gradual changes in the sites of auxin production during leaf development, due to the control mechanism of *leaf apical dominance* (Aloni 2001), result in organized patterns of reticulate xylem and phloem veins in *Arabidopsis* leaves. In a young leaf primordium, auxin can be produced in almost every cell, but only an orderly pattern of auxin production can organize leaf shape and define a well-designed vascular network (Aloni et al. 2003; Aloni 2024). The mechanism controlling auxin production in a growing leaf primordium organizes leaf’s basipetal development (occurring from leaf’s tip to petiole) and the hierarchy of its vein pattern, size and chronologic development, which characterize leaves.

**Fig. 1:**
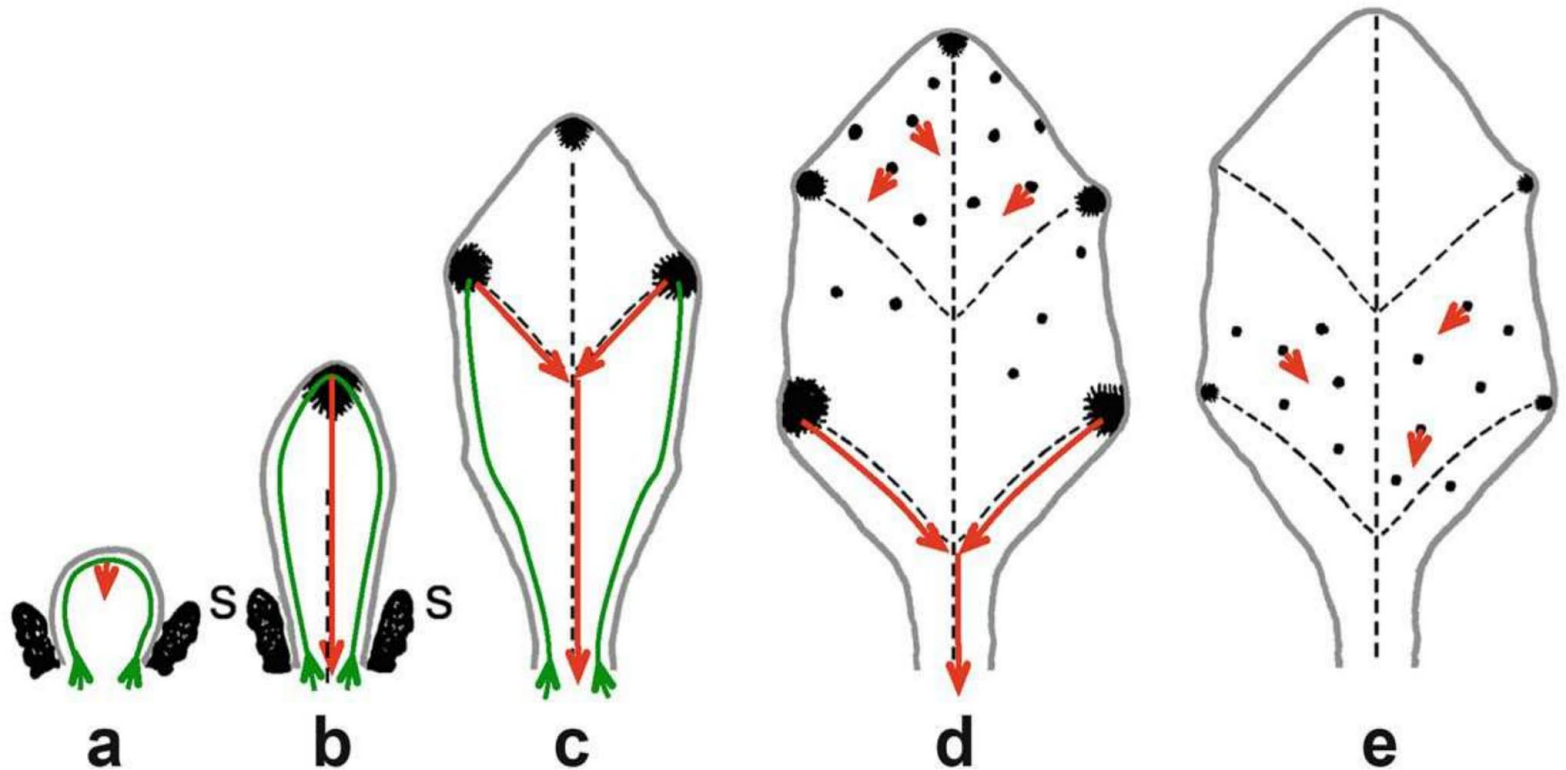
Schematic diagrams showing the gradual changes in sites (black spot locations) and concentrations (black spot size) of high vs. low-auxin production during leaf primordium morphogenesis in *Arabidopsis*. The rising green lines illustrate the upward polar auxin flow through the differentiating epidermis during the early stage of primordium development (**a**–**c**), originating from nearby auxin-producing young leaves. The red arrows show the experimentally confirmed directions of the downward (basipetal) vein-inducing polar auxin movement, descending from the differentiating hydathodes in the growing tip and lobes (**b**–**d**). The midvein in b is drawn as broken line, which matures upward, acropetally (due to the auxin accumulation above the short cells of the future abscission layer), although it is induced by the basipetal (downward) polar IAA flow (red arrow) descending from the primordium tip (**b**). Short red arrows in the lamina that originate from small black spots indicate random possible auxin flow directions from minor auxin production sites (**d**, **e**), which induce the tertiary veins and freely ending veinlets. The ontogeny of the midvein and secondary veins is illustrated by broken lines (marginal and minor veins are not shown). **a** Early high auxin production occurs only in the stipules (s) of a very young leaf primordium, before free auxin is detectable in the tip. **b** Auxin maximum production in the tip of a fast-elongating primordium induces acropetal midvein differentiation, illustrating “leaf apical dominance”. **c** Auxin maxima production in the fast-expanding upper lobes induces the upper secondary veins and matures into hydathodes. **d** Auxin maxima production in the lower lobes inducing the lower secondary veins. These lobes later mature into hydathodes; randomly distribution of minor auxin production sites starts first in the upper lamina (**d**), and later also in the lower lamina (**e**), induces the tertiary veins and freely ending veinlets, during later phase of primordium development (from Aloni 2024)

Although there are cases in which the sites of auxin production during leaf development are not clear (Scarpella 2024), we observed a general basipetal pattern of gradual shifts in the sites and concentrations of free-auxin production in *Arabidopsis* leaves, occurring first as the strongest auxin maximum in the tip of a leaf primordium, then gradually progressing downward as auxin maxima along the margins, and finally appearing as minor low-auxin producing sites in the central regions of the lamina (Fig. 1) (Aloni et al. 2003).

**Fig. 2:**
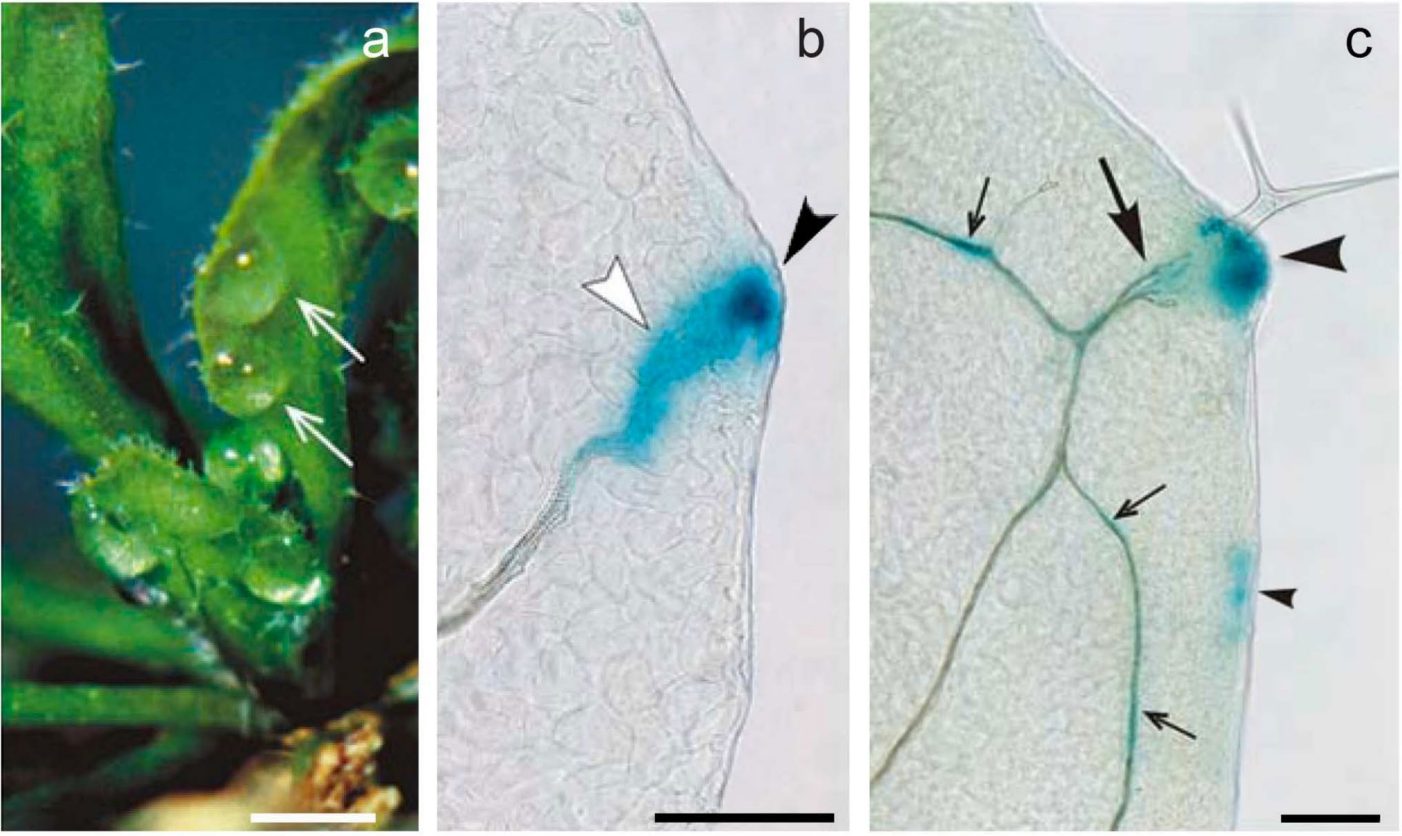
Leaf hydathodes, **a** their guttation, **b** differentiation of their supporting secondary strand, and **c** development of their marginal freely ending vessels. **a** Early morning guttation of an outdoor *Arabidopsis thaliana* plant, showing typical secretion of water droplets (*arrows*) below the hydathodes of young leaves. Micrographs (**b**, **c**) of cleared leaf primordia of DR5::GUS transformed *A. thaliana* demonstrating how a high-auxin concentration induces a secondary strand (**b**), below a developing hydathode (**c**), visualizing the process by the blue staining of *DR5::GUS* expression at the lobe of a young leaf. **b** Showing a center of strong expression (*black arrowhead*) marking the synthesis site of high-auxin concentration, from which the auxin starts to flow downward in a diffusible pattern that gradually becomes canalized (*white arrowhead*) to a narrow stream which induces the vascular strand. **c** *DR5::GUS *expression at the margin (*large arrowhead*) in a more developed hydathode with four freely ending vessels (*large arrow*) differentiating toward the margin. Note some weaker blue staining near the margin (*small arrowhead*) and *GUS* expression within the vascular strands (*small arrows*). Bars = 1 mm (**a**), 150 µm (**b**), 250 µm (**c**) (from Aloni et al. 2003)

The auxin maximum of the leaf’s tip induces the midvein, while the peripheral auxin maxima induce the large secondary veins. Importantly, all these peripheral auxin maxima in the tip and lobes are differentiating hydathodes, which would start morning guttation (Fig. 2a) when the leaf matures (Aloni et al. 2003). The auxin maxima in differentiating hydathodes (Fig. 2b, c) induce well-developed major vascular strands that support guttation activity. Recently, it was experimentally shown that the upper auxin maxima at the leaf periphery inhibit auxin production below them, consequently suppressing hydathode development and decrease hydathode size in the lower lobes (Aloni 2021).

Only when the inhibiting auxin-maxima producing sites along the periphery stop auxin production, then the minor low-auxin-producing sites inside the lamina become active and start to induce the free-ending veinlets and delicate high-order (tertiary) veins that connect to the early induced secondary veins (Aloni et al. 2003; Aloni 2021).

The basipetal pattern of gradual shifts in free-auxin production demonstrate a unique type of dynamic *apical dominance* control mechanism of leaves, where the tip’s high-auxin production inhibits auxin production below it for a short duration. As the leaf tip matures relatively fast its high-auxin production declines, then the free-auxin production increases in the upper lobes (which are not inhibited anymore by the tip), and they start to become the auxin maxima-inhibiting sites (Aloni et al. 2003). This process extends gradually downward from the tip to the base of the leaf primordium, as was hypothesized by the *leaf-venation hypothesis* (Aloni 2001).

## Free and conjugated auxins in leaves and flowers

Interestingly, it should be mentioned that both the youngest leaf and flower primordia are already loaded with conjugated auxins (Aloni et al. 2003, 2006a) that are inactive as hormonal signals. These bound auxins detected by immunolocalization with specific polyclonal antibodies, as was shown with polyclonal and monoclonal antibodies in *Arabidopsis* leaves and flowers (Aloni et al. 2003, 2006a), serve as a reservoir from which the free auxin can be released (Aloni 2021). These conjugated auxins result from the local accumulation of the upward free-auxin flow, through the differentiating epidermis, originating in adjacent young leaves (Reinhardt et al. 2003; Benková et al. 2003; Scarpella et al. 2006). Accordingly, the youngest leaf and flower primordia likely start as sinks for auxin and become sources of the hormonal signal during later stages of primordium development (Aloni et al. 2003; Aloni 2021), either by hydrolysis of conjugated auxin, or by local auxin synthesis occurring primarily in the developing hydathodes (Aloni et al. 2003; Baylis et al. 2013; Yagi et al. 2021). Due to the vital importance of constantly producing the primary shoot signal, i.e., IAA, this strategy of plants to maintain a pool of bound auxin in the upper shoot organs prevents situations of free-auxin deficiency (Aloni 2021).

## Vascular differentiation: description vs. control mechanism

Experimental evidence shows that differentiating hydathodes, the water secreting glands (during morning guttation) at the leaf periphery, are the primary sites of auxin production during leaf morphogenesis (Aloni 2001). Molecular evidence confirms and demonstrates that incipient hydathodes are the main sites of high-auxin biosynthesis during leaf development (Baylis et al. 2013; Yagi et al. 2021), which are likely promoted by cytokinins (CKs) from the root tips (Aloni et al. 2006b). The differentiating hydathodes induce the major vascular strands, namely, the midvein and the secondary veins (Fig. 2b).

Scarpella et al. (2006, 2010) describe how during leaf initiation, epidermal auxin flow converges to form a local high-auxin activity site at the tip of the primordium. From this PIN-FORMED1 (PIN1) convergence point in the epidermis, auxin is transported from the epidermal layer into internal tissues, where it induces the formation of a vascular strand, suggesting the *epidermal auxin-focusing mechanism* for major-vein positioning (Scarpella et al. 2006). After primary morphogenesis of the midvein and secondary strands, when the basic leaf and vasculature patterns are already formed, high-order (tertiary) delicate veins appear in continuity with pre-existing vasculature in the expanding blade. The high-order veins can end freely in the lamina, or become connected to early induced strands. However, this detailed description does not explain the general auxin mechanism that controls the gradual basipetal orderly pattern of the vein system in leaves. For example, it does not explain how the upper differentiating hydathodes inhibit the lower hydathodes, inducing the polar downward pattern of auxin maxima development, and why there is a delay in the initiation of the delicate high-order veins in the lamina. Also, why an auxin efflux inhibitor, prevents the formation of high-order veins and freely ending veinlets in the lamina (Scarpella et al. 2006; Wenzel et al. 2024); but these patterns are explained by the *leaf apical dominance* mechanism (Aloni 2001; Aloni et al. 2003), namely, that the auxin efflux inhibitor does not allow drainage of IAA downward, thus causing a continued high-auxin concentration at the upper margins of the leaf, which inhibits low-auxin initiation inside the lamina (shown in Aloni 2021).

Our leaf paper (Aloni et al. 2003) visualizes how a high-concentration of auxin produced in a lobe starts to move by diffusion and gradually become canalized in a downward polar movement forming a vascular strand (Fig. 2b), demonstrating and supporting Sachs’ *canalization hypothesis* (Sachs 1981). A similar developmental pattern indicating auxin canalization was observed with the *MONOPTEROS* (*MP)* gene by Wenzel et al. (2007). Nevertheless, experimental evidence indicates that vein patterning features are best accounted for by a combination of the polar auxin transport, facilitated auxin diffusion through plasmodesmata intercellular channels (Scarpella 2024).

## Tapetum in developing anthers synchronize flower biology

The tapetum cells in the anthers produce high-auxin concentrations (Aloni et al. 2006a). This phenomenon was later confirmed by Cecchetti et al. (2017). Aloni et al. 2006a) found that the tapetum cells supply the developing pollen grains with free auxin that accumulates in the pollen grains as conjugated auxins. When the mature pollen grains with their accumulated conjugated auxins germinate on the stigma of the gynoecium, they start to hydrolyze free auxin, which promotes the rapid intrusive growth of their pollen tubes to the embryo sac. This indicates that auxin is likely involved in the mechanism that controls the growth of the pollen tube with the male gametophyte to the egg cell in the ovule.

**Fig. 3:**
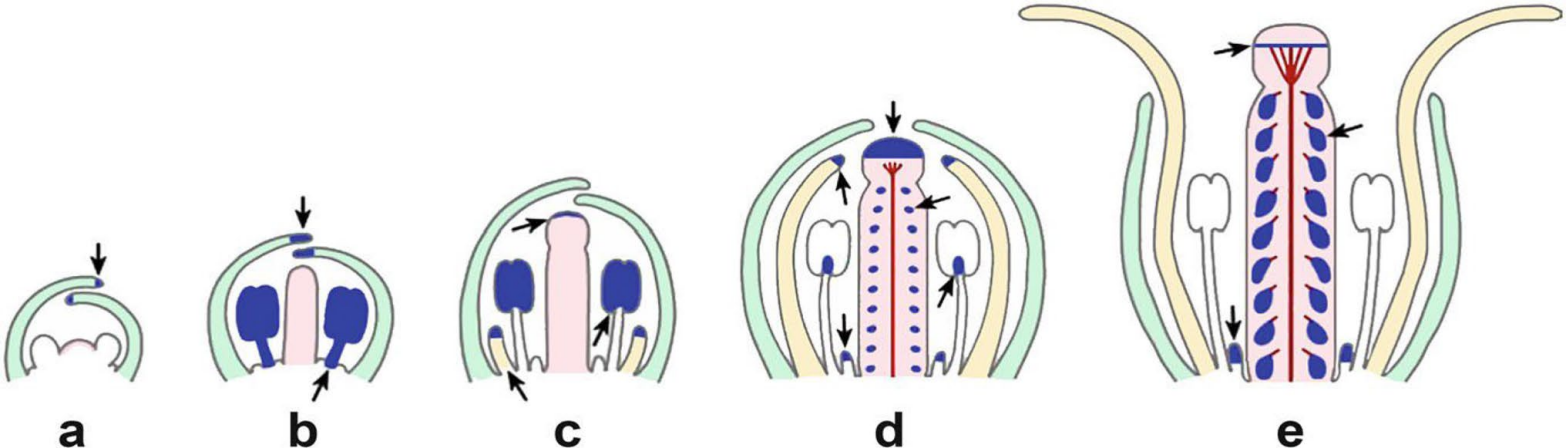
Schematic diagrams showing the gradual changes in sites (blue spot locations) and concentrations (blue symbol sizes) of IAA production (detected by *DR5::GUS* expression) during *Arabidopsis* flower and early fruit development. Arrows mark sites of auxin production starting at the tip of floral organs during their development (**a**–**e**) and at the ovules and developing seeds in the gynoecium (**d**, **e**). The ontogeny of the gynoecium midvein, characterized by its wide fan xylem induced by IAA descending from the stigma (**d**, **e**) and the short xylem veinlets induced by developing seeds are illustrated by red lines (**e**). **a** Young floral bud with incipient IAA production at the tip of the sepals [the bud is loaded with conjugated auxin (Aloni et al. 2006a)]. **b** IAA production at the sepal tips and massive bioactive auxin production in the stamens, demonstrating “stamen dominance” characterized by complete petal suppression. **c** Decreased free-auxin production in the stamens (DR5::GUS activity limited to the anthers) is followed by incipient auxin production in the growing petals and stigma. **d** High IAA production in the stigma; low-auxin production in the ovules, the nectaries, the petal tips and stamen-filament tips. **e** Residual IAA production beneath the stigma, elevated auxin production in developing seeds, and continuous production in nectaries (from Aloni et al. 2006a)

Selective removal of floral organs that altered flower development and free-auxin production demonstrated that the anthers, which are the highest auxin-producing organs, inhibit petal elongation and nectar-gland activity until pollen grain maturity, which synchronize flower organ development and optimize flower pollination biology (Aloni et al. 2006a). This developmental pattern during flower development (Fig. 3) demonstrates the importance of the *apical dominance* concept (Thimann and Skoog 1933) as a basic auxin mechanism, where flower organs that produce high-auxin concentrations inhibit neighboring organs, thus regulating whole flower development; explaining the observation of Bowman et al. (1989) why the early induced petal primordia in *Arabidopsis* are inhibited and do not grow until stage 9, which is a general phenomenon found also in other flowering plant species (Endress 1994).

Our both studies stimulated us to extend the idea of *apical dominance* to roots as well, where the active dominant root tip that produces the highest amounts of the cytokinin hormone suppress and delays lateral root development, giving priority to the primary root in competition to reach deep water, thus optimizing root growth (and plant survival in drying environments) and shaping its architecture (Aloni et al. 2006b; Aloni 2021).

## Phytohormone crosstalk

Auxin is the primary hormonal signal that determines organ developmental patterns in shoots. Auxin regulates organ development in balance with other hormonal signals, mainly CKs and gibberellins (GAs). These phytohormone crosstalks regulate organ and whole plant development. CKs promote cell divisions at the shoot apical meristems, leaf development and expansion in various regions of the leaf, especially at the margins (Wu et al. 2021; Navarro-Cartagena and Micol 2023) where the auxin maxima occur (Aloni et al. 2003), thus collectively they determine the final morphology of leaves. CKs also play major roles in flower development, specifically during gynoecium and fruit morphogenesis, including a role in valve margin formation in *Arabidopsis* siliques (Marsch-Martínez et al. 2012; Marsch-Martínez and de Folter 2016).

Gibberellins promote leaf and floral organ elongation (Ritonga et al. 2023), which is important for organ expansion, and in flower biology, especially for pollination (Gastaldi et al. 2020). Like IAA, the GA flows in the vascular strands and is accumulated at the leaf junction (Dayan et al. 2012). In addition, GA is the specific signal that induces fibers in shoots, in both leaves and flowers. Importantly, GA does not induce fiber differentiation in the absence of auxin (Aloni 2024).

## Discussion

A detailed description is an important step in the discovery of a new biologic phenomenon. However, to understand how this phenomenon is regulated, its control mechanism should be elucidated. Here, I have focused on the *leaf apical dominance* control mechanism (Aloni et al. 2003), *vs.* the detailed description of the *epidermal auxin-focusing mechanism*, observed with the PIN1 protein (Scarpella et al. 2006), which together form a general understanding of how the leaf vascular system is hormonally controlled.

In addition, we (Aloni et al. 2006a) explained the auxin control mechanism that synchronizes flower development by high-auxin-producing tapetum cells in developing anthers, which inhibit petal growth and nectar-gland activity until pollen grain maturation. A decade later, Cecchetti et al. (2017) published a study on high-auxin concentration in developing tapetum cells of *Arabidopsis* flowers, proposing that the high-auxin concentration produced by the tapetum is necessary to ensure correct and coordinated pollen maturation, as well as anther development. However, they failed to mention how the anthers—due to their inhibiting high-auxin concentration—synchronize the biology of the *whole* flower by a control mechanism explained and supported by the pioneering findings in Aloni et al. (2006a), as mentioned above.

Our leaf (Aloni et al. 2003) and flower (Aloni et al. 2006a) papers clarify that the auxin control mechanism known as *apical dominance* proposed by Thimann and Skoog (1933) is still relevant today for understanding and explaining developmental patterns induced in leaves, flowers, and roots (Aloni et al. 2006b).

Importantly, developmental patterns parallel to those induced by auxin were later discovered for cytokinin (Marsch-Martínez et al. 2012) and GA (Dayan et al 2012; Ritonga et al. 2023). Although auxin is the primary signal inducing the organized patterns mentioned above, the developmental patterns of the other hormones demonstrate local crosstalk and interactions between these key signals, in both leaves and flowers.

## Data Availability

Data available upon request from the author.
